# Evaluation of stress factors among the elderly in the nursing homes for the elderly (Eram and Mother) in Kermanshah, in 2015

**Published:** 2015

**Authors:** Z Moradi, M Far Ajallah Bike Nouri, M Mohammadi, F Esfandnia, P Taovsi, A Esfandnia

**Affiliations:** * Isfahan University of Medical Sciences, Isfahan, Iran,; ** Tabriz University of Medical Sciences, Tabriz, Iran,; *** Kermanshah University of medical sciences, Kermanshah, Iran

**Keywords:** assessment, stress factors, elderly

## Abstract

**Introduction:** The goal of this research was to assess the stress factors among the elderly living in nursing homes (Eram and Mother) in Kermanshah, in 2015.

**Research method:** This was a descriptive - cross analysis and was performed in the first half of 2015 in a sectional way. The statistical population included 150 elderly men and women aged 60 to 74, and the sample size was selected from 108 people using the Cochran formula. A standard questionnaire was used to collect data from a previously validated survey. Finally, a total of 100 questionnaires were filled in and data were analyzed by using Amos 21 and SPSS 21 software.

**Results:** The results revealed that the dimension of the physiological problems had the highest average and variance of 5.36 ± 21.02 and Disappointment, Home empty, Disability and independence, Relationship problems, Seclusion with an average and variance of 3.12 ± 20.55, 5.29 ± 18.82, 4.54 ± 17.72, 3.59 ± 16.66 and 4.55 ± 16.41, had the largest average and variance.

**Conclusion:** Given that the greater number of elderly live with the family in Kermanshah and have sufficient support, recommended that the government planned to reduce isolation and increase the social support for this group of elderly nursing.

## Introduction

The increasing population of the aged is one of the most significant economic, social, and health challenging in the current century [**1**]. Based on UN estimates, the world’s elderly population of 350 million people reached one billion in 1975 and it is estimated to reach one hundred million people in 2025, the growth of the elderly population growing much faster than the total population of the world, most of them living in developing countries [**2**]. So, an increasing age increases the risk of one or more chronic diseases so that most humans above 60 years old have at least one chronic disease [**3**]. Almost 80 percent of the elderly patients had a chronic disease so that they became more vulnerable than others [**4**]. Aged people are more exposed to various diseases [**5**]. The physiological changes that occur during the aging process and certain changes in the nervous structure and the musculoskeletal structure could be concerned regarding the act of the gestures, increasing the risk of accidents such as burns, accidents, etc. [**6**]. One of the goals of dynamic aging is to reduce disability related to chronic diseases in old age [**7**]. According to the National Council of the Elderly, persons over 60 years, are elderly [**8**]. With regard to improving health and economic communities, each year, the number of elderly increases [**9**]. At the equal time, almost seventy percent of the elderly suffer from multiple chronic diseases. Due to the growing count of elderly in the population and the increase of chronic diseases among older people with various chronic conditions, their care can be difficult and, unfortunately, care systems act inefficiently [**10**]. To improve the control population and increase life expectancy, as well as improve the treatment, the global population ages [**11**]. The increase of the elderly population in advanced countries is over and now further than fifty percent of the world's elderly live in developing nations. Based on the UN report, in 2000, the elderly people in developing countries was 12.5%-17% of the world’s people [**12**]. On the other hand, the total number of elderly in the world, in 2006, was 687 million and will be of 923 thousand in 2050, and the figure reached one billion and 968 million and 53 thousand people, also, according to the report, currently 6% of the population aged up to 26 million and 393 thousand (equivalent to 26% of the population per year). Also, according to the center’s report, the life expectancy for men and older women in Iran during the years 2005 and 2010 was 77 and 78 years respectively [**13**]. Taking into account the tressing factors period, aging is a loss, the loss of a child, spouse, vision, hearing, occupation, social status, etc. [**14**]. Elderly people, who are prone to disease and disability, are physically different regarding the mental health of those whom they accept. Some psychological problems within this period of life are more prevalent [**15**]. The aging population influences the various economic and political aspects, causing a sharp rise in public spending and imposing additional pressure on social security [**16**]. One of these difficulties is stress. Stress and new diseases of the civilization today is the rise of many physical and mental diseases [**17**]. The aim and principles of this research were to evaluate the stressor factors among the elderly living in nursing homes for the elderly (Eram and Mother) in 2015, in Kermanshah 2015.

## Method

This cross-sectional analytical-descriptive research design was done in the initial half of 2015 in the Welfare Rehabilitation Center in Kermanshah to evaluate the stressor factors among the elderly, in the nursing houses for the elderly (Eram and Mother). The statistical community included all 60 years old people and older men and women who were staying in welfare institutions. The sample included 150 elderly men and women aged 60 to 74, and a sample size of 108 Cochran formulas was done, which came to a final number of 100 questionnaires filled in. The surveys were divided randomly. Data collection was conducted by using a standard conventional questionnaire. To collect the questionnaires, the Elderly Rehabilitation Center for Men (Eram) and Women (Mothers) were visited and a list of the elderly people was provided. Then, the forms were filled and the final data were analyzed with the aid of SPSS 21 and AMOS 21 software. A proven and reliable questionnaire from the research of Sadrossadat et al. (2013) [**18**] was approved. Cronbach’s alpha method of total scale was equal to 0.95 and the Spearman–Brown’s was 0.84 and 0.79 respectively, the tests indicating a good reliability for this scale [**19**].

## Results

The results revealed that in terms of gender, 53% were females (53 patients) and 47% (n = 47) were males. 33% were single (late spouse and widow), 33 and 67 percent (67 people) were married. 33% (32) had an under diploma certificate, 34% (33) held a diploma, 11.3% (11) held an associate degree, 16.5% (16) had a baccalaureate and 5.2% (5 patients) held an MA and upper. 

**Table 1:** Mean, variance and the mean scores by applying the Friedman examination

Dimension	Mean Rank	Mean	Std. Deviation
Disappointment	2.61	16.4100	4.55958
Seclusion	4.57	20.8500	3.12169
Home empty	2.77	16.6600	3.59073
Relationship problems	3.54	18.8200	5.29604
Physiological problems	4.26	21.0200	5.36728
Disability and independence	3.26	17.7200	4.54402

The table above shows the dimension of the physiological problems with the largest mean and variance of 5.36 ± 21.02, Disappointment, Home empty, Disability and independence, Relationship problems, Seclusion with a mean and variance of 3.12 ± 20.55, 5.29 ± 18.82, 4.54 ± 17.72, 3.59 ± 16.66 and 4.55 ± 16.41, was the highest average and variance. Also, the rankings of the Friedman test with chi-square 91.092 and, df = 5, and the significant level of 000 was used, the results showing the dimension Seclusion, with a medium grade of 4.57, the highest rank and aspects of Physiological problems and Relationship problem and Disability Disappointment and independence and Home empty and the average rating respectively 4.26, 3.54, 3.26, 2.77 and 2.61, having the greatest rank. 

**Table 2:** Relationship between stressor factors and their dimensions

			Estimate	S.E.	C.R.	P
Disability and independence	<---	Stressors of aging	.157	.023	6.878	***
Physiological problems	<---	Stressors of aging	.168	.028	5.974	***
Relationship problems	<---	Stressors of aging	.175	.027	6.474	***
Home empty	<---	Stressors of aging	.104	.019	5.422	***
Seclusion	<---	Stressors of aging	.112	.015	7.294	***
Disappointment	<---	Stressors of aging	.196	.020	9.978	***
***Indicates the importance of the relationship at the level of 95%.						

According to the table, there is a meaningful relationship within the dimensions of stress factors among the elderly living in nursing homes for the elderly (Eram and mother) in Kermanshah. The means confidence level is smaller than 0.05, meaning the relation within the dimensions of significant stress factors and positive factors.

**Table 3:** Total directly or indirectly standardized effects

Standardized Total Effects		Standardized Direct Effects	Standardized Indirect Effects
	Stressors of aging	Stressors of aging	Stressors of aging
Disappointment	.708	.708	.000
Seclusion	.591	.591	.000
Home empty	.478	.478	.000
Relationship problems	.545	.545	.000
Physiological problems	.515	.515	.000
Disability and independence	.569	.569	.000

The table above shows that the standard of the total amount of their direct effects is equal to the dimensions Disappointment, Seclusion, Home empty, Relationship problems, Physiological problems, Disability and independence equal to the amount 0.708, 0.591, 0.478 , 0.545, 0.515 and 0.569 respectively. Also indirect effects are standard for all resolution 0.000, respectively.

**Table 4:** The final model-fitting index of the study

Acceptation level	Obtained value	Interpretation	Accepted level	Standard criterion
Accepted	283.483	Chi-square achieved compared with the chi-square table for a particular degree of freedom.	Value of chi-square in the table	Chi-square CIMIN
Accepted	.042	Should be less than 0.5	Less than 0.5	The root mean square error of estimate (RMSEA)
Rather Accepted	.854	Near to 0.95 is fitted	Zero (non-fitted), one (good fitted)	Louise Tucker TLI
Accepted	4.899	Smaller than 1 shows weakness; larger than 5 shows the need to improve fitness levels.	1 to 5	relative Chi-square CMIN/ DF
Rather Accepted	0.575	Should be more than 0.5 or 0.6		Normalized frugal fit index PNFI
Rather Accepted	0.589	Should be more than 0.5 or 0.6		Comparative frugal fit index PCFI
Rather Accepted	0.848	Should be more than 0.9	Comparing the model to model without its relationship	Bentley Bonet index NFI normalized Fitness indicator
Rather Accepted	.883	Should be more than 0.9	Examining the model with model beyond its relationship	CFI
Rather Accepted	.878	Standard value is more than 0.9	Between 0 and 1	incremental Fitness index IFI
Chi-square = 283.483				
Degrees of freedom = 15				
Probability level = .000				

The rate of the economy or PRATIO, a sort of moderate fit indices admitted in itself, is not fit index, but slightly shows the amount of which the analyst has used the description of free parameters. This index was produced according to the degrees of freedom model, which can reach independence, an amount within zero and the one to get any size is very smaller, demonstrating that the researcher has used more money in the free parameters. Often, larger values of 0.5 for this sign have seen that this rate is of 0.714. Also, for an enough amount of samples, the HOTLTER index used in this research specimen number 41 was acceptable and according to the research specimen size it was of 100 companies and designs of these indicators were also fitted. Indicators ECVA, MECVI, AIC, BCC, were used to discover the most balanced model deemed and a model with the least quantities to more fine models were recognized in this study, respectively, 3.303, 3.268, 326.999, 323.483, which amounted to 3.268, ECVA being the leading efficient model. 

**Fig. 1:**
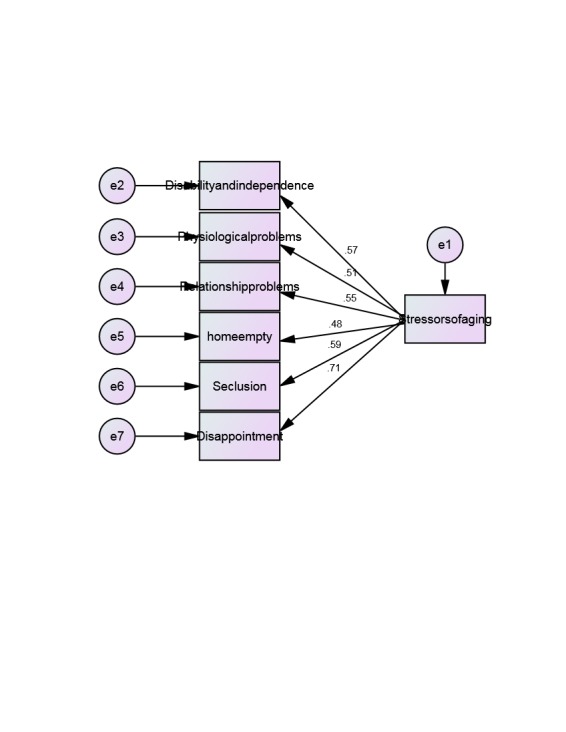
ECVA model

Based on the above, stress factors have a direct effect on dimension Disability and independence (0.57), Physiological problems (0.51), Relationship problems (0.55), Home empty (0.48), Seclusion (0.59) and Disappointment (0.71). The highest impact between the stress factors among the elderly and those dimensions related to dimension Disappointment was at a rate of 0.71 percent and the lowest for Home empty dimension 0.48 respectively.

## Discussion and Conclusion

The goal of this research was to evaluate the stress factors among the elderly in Kermanshah. Based on the results achieved, the dimension physiological difficulties, with the greatest average variance dimensions of 5.36 ± 21.02 respectively and then Disappointment, Home empty, Disability and independence, Relationship problems, Seclusion, with an average and variance of 3.12 ± 20.55, 5.29 ± 18.82, 4.54 ± 17.72, 3.59 ± 16.66 and 4.55 ± 16.41, was the highest average and variance. Oldehinkel et al. noted that physical health problems, problems related to disability, hearing, vision, and memory as stressor for seniors, were medical conditions due to a decreased ability to carry out their job and in severe cases caused stress to others in the association with older people [**20**]. Difficulty in remembering and recalling everything, reducing the power of the senses (hearing, smell, touch), bladder control issues, specific diseases, changes in sleep, changes in diet, inability to perform daily activities (shopping, food preparation, cleaning the house), are factors that the elderly people are exposed to. Research results of Shiri Mohammad Abadi et al. (2014) showed that the events related to financial issues, social issues, labor issues, health and mortality in the elderly, were stressful [**18**]. Research results of Zia Pour and Kianpour (2014) showed that 44% of the elderly people have sleep problems, 23% hearing problems, 63.6% vision problems, 21.7% constipation, 45.3% memory impairment, 14.8% urinary tract problems, 90.8% dental problems, and 43% stress [**15**]. For the ranking dimensions of stress factors among the elderly, Friedman test with chi-square df =91.092 5 was used, and the significance level was sig = 0.000 results, showing that dimension Seclusion, with an average grade dimensions of 4.57 had the highest rank and then Physiological problems and Relationship problem and Disability and independence and Home empty and Disappointment with the average rate, respectively, 4.26, 3.54, 3.26, 2.77 and 2.61, were the leading ranks. Galligan stated that one of the very significant aspects in old age is to keep or create stronger ties and participate in social activities. Loneliness and limited social relations are a significant cause of stress in the elderly. In general, powerful social connections cause social support and empowerment of people to help others in crisis [**21**]. In the Barry study, approximately 60% of the cost of health care for the elderly was shown to statement for 35% of the medical evacuations and 47% of the days in the hospital to be included [**22**]. Given that the majority of elderly people existing in Kermanshah with the family and enjoying a sufficient support from the government suggested that programs are created to reduce isolation and increase the social support for this group in elderly nursing.

**Acknowledgment:**

Colleagues of the Medical Sciences, who assisted us in implementing this project. This project did not have a source of funding. 
